# Editorial: Characterizing the uncharacterized human proteins

**DOI:** 10.3389/fgene.2023.1203691

**Published:** 2023-05-04

**Authors:** Yan-Ming Xu, Mee-Hyun Lee, Chi-Ming Wong, Andy T. Y. Lau

**Affiliations:** ^1^Laboratory of Cancer Biology and Epigenetics, Department of Cell Biology and Genetics, Shantou University Medical College, Shantou, Guangdong, China; ^2^ College of Korean Medicine, Dongshin University, Naju, Republic of Korea; ^3^ Department of Health Technology and Informatics, The Hong Kong Polytechnic University, Hong Kong SAR, China

**Keywords:** uncharacterized human proteins, proteoforms, open reading frame (orf), alternative splicing, genomics, proteomics, systems biology

As of the first week of April 2023, according to the data from the UniProt Knowledgebase (https://www.uniprot.org) (UniProt Consortium, 2023), the human proteome has a record of 20,422 canonical as well as 21,998 non-canonical protein isoforms. These figures are noteworthy because the number of non-canonical isoforms is almost equal to the number of canonical ones, suggesting that “non-canonical” isoforms may be equally important as their canonical counterparts. Despite the fact that scientists have studied canonical isoforms in greater detail over the years, there are still hundreds to thousands of uncharacterized canonical and non-canonical isoforms as their biological functions are yet to be revealed. Besides, the discovery of protein encoding ncRNAs (Jackson et al., 2018) has added another layer of complexity to the situation, and as such, the current reported total number of human proteoforms (Smith and Kelleher, 2018) is likely to be underestimated. The Research Topic titled “*Characterizing the uncharacterized human proteins*” showcases the latest discoveries in the field of unstudied or under-studied proteins/isoforms. It consists of 9 published manuscripts, including 6 research and 3 review articles, with 66 different contributors. This Research Topic covers a wide range of studies, from individual uncharacterized proteins to high-throughput analyses of novel peptides/protein variants in both normal and pathological conditions.

Among the research works covered in this Research Topic, Tan et al. unveiled the potential relationships between essential micronutrients and uncharacterized human proteins C9orf85 and CXorf38, where their expressions could be selectively induced by manganese and selenium. Dong et al. demonstrated that Max interacting protein 1–0 (Mxi1-0), a functional isoform of Mxi1, potentiates hypoxic pulmonary hypertension through MEK/ERK/c-Myc-mediated proliferation of pulmonary arterial smooth muscle cells. Tian et al. reported on a human case carrying two rare variants of ARHGAP31 [c.2623G>A (*p*.Glu875Lys)] and FBLN1 [c.1649G>A (*p*.Arg550His)], where the synergistic effects of these two protein mutants may potentiate the terminal transverse limb defects (TTLD), expanding the clinical complexity of mutant gene product interactions in genetic disorders. Zhang et al. studied arrestin domain containing 2 (ARRDC2), a protein in the *α*-arrestin family, in ovarian cancer (OC). They found that high ARRDC2 expression level is associated with malignant biological behavior and poor overall survival of OC, suggesting that ARRDC2 can be used as a potential indicator to evaluate the prognosis of OC. Chen et al. used an untargeted proteomic approach with LC-MS/MS to screen and functionally analyze peptides from the placenta of healthy subjects versus patients with preeclampsia (PE). They identified a differentially-expressed peptide named placenta-derived peptide (PDP, with the sequence AASAKKKNKKGKTISL), derived from the precursor protein eukaryotic translation initiation factor 4B, which could bind to TGF-β1 and impact the Smad signaling pathway, demonstrating that placental bioactive peptides may regulate placental function during the progression of PE. Wu et al. used translatome sequencing to investigate alternative splicing (AS) isoforms in human hepatocellular carcinoma MHCC97H cell line. They identified 50 novel protein isoforms in mass spectrometry datasets, demonstrating the potential of translatome sequencing in investigating the proteome of AS isoforms.

Furthermore, this Research Topic includes excellent literature reviews. Li and Kalev-Zylinska provided a comprehensive and up-to-date summary of molecular alterations in myeloid leukemia associated with Down syndrome, including the aberrant expression of proteins on chromosome 21 (such as C21orf66) and GATA1 mutations, which drive expression of a truncated GATA1 protein. Chiang et al. focused on reviewing a lysine methytransferase named SETD7 (also known as KIAA1717). Besides its well-known epigenetic regulatory role as a histone lysine methyltransferase, SETD7 can also methylate other nonhistone proteins (over 30 substrates, including transcriptional-related proteins and enzymes). Thus, manipulating SETD7 and subsequently its substrate methylation levels might be possible strategies for cancer intervention. Lastly, Yang et al. discussed the YT521-B homology domain family proteins [YTHDFs, including YTHDF1 (also known as C20orf21), YTHDF2 and YTHDF3], in which more and more studies have supported these N6-methyladenosine readers could play a key role in tumor transcription, translation, protein synthesis, tumor stemness, epithelial−mesenchymal transition, immune escape, and chemotherapy resistance.

**FIGURE 1:**
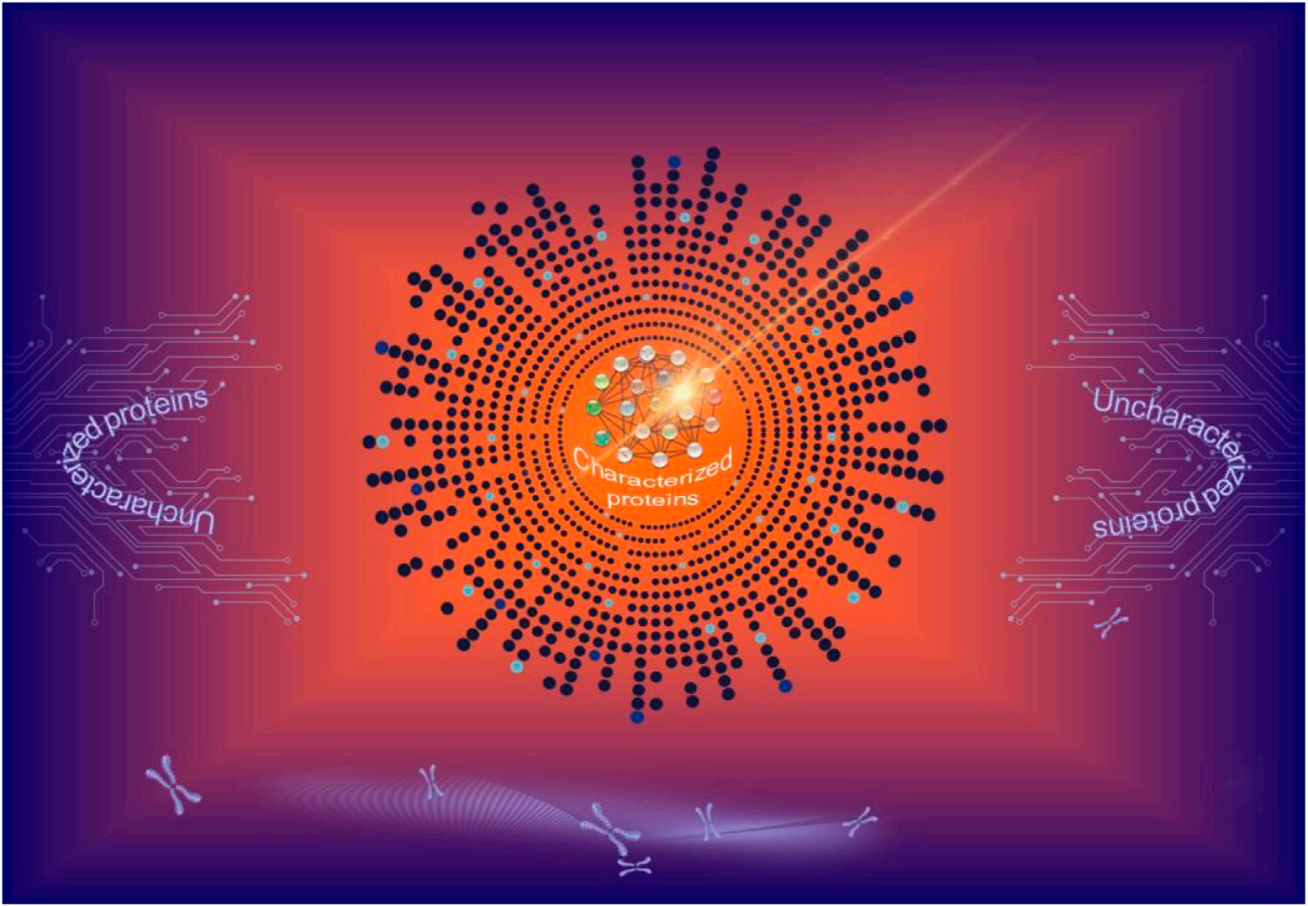
The exploration of the uncharted territories of the human proteome. As time goes on, all uncharacterized human proteins will gradually be characterized and merged to the existing protein network.

Although much more collaborative efforts are required to unveil the mysteries of the cellular functions of uncharacterized human proteins, the works in this Research Topic would certainly arouse the attention of scientists in the urgent need to fill in the knowledge gaps in this frontier in the years to come. With this goal being set, as time goes on, sooner or later all uncharacterized human proteins would ideally be characterized and have their names rewritten in history (Figure 1).
